# Vertical distribution of aerosols in dust storms during the Arctic winter

**DOI:** 10.1038/s41598-019-51764-y

**Published:** 2019-11-06

**Authors:** Pavla Dagsson-Waldhauserova, Jean-Baptiste Renard, Haraldur Olafsson, Damien Vignelles, Gwenaël Berthet, Nicolas Verdier, Vincent Duverger

**Affiliations:** 1 Agricultural University of Iceland; Faculty of Environmental Sciences, Hvanneyri, Borgarnes, IS 311 Iceland; 2 Czech University of Life Sciences Prague, Faculty of Environmental Sciences, Department of Ecology, Prague, 160 00 Czech Republic; 3 LPC2E-CNRS, 3A avenue de la recherche scientifique, 45071 Orléans cedex 2, France; 4 University of Iceland, Department of Physical Sciences, Reykjavík, IS 101 Iceland; 5 Icelandic Meteorological Office, Reykjavik, Iceland; 6 Centre National d’Etudes Spatiales, 18 avenue Edouard Belin, 31055 Toulouse cedex, France

**Keywords:** Experimental particle physics, Atmospheric chemistry

## Abstract

High Latitude Dust (HLD) contributes 5% to the global dust budget, but HLD measurements are sparse. Dust observations from Iceland provide dust aerosol distributions during the Arctic winter for the first time, profiling dust storms as well as clean air conditions. Five winter dust storms were captured during harsh conditions. Mean number concentrations during the non-dust flights were <5 particles cm^−3^ for the particles 0.2–100 µm in diameter and >40 particles cm^−3^ during dust storms. A moderate dust storm with >250 particles cm^−3^ (2 km altitude) was captured on 10^th^ January 2016 as a result of sediments suspended from glacial outburst flood Skaftahlaup in 2015. Similar concentrations were reported previously in the Saharan air layer. Detected particle sizes were up to 20 µm close to the surface, up to 10 µm at 900 m altitude, up to 5 µm at 5 km altitude, and submicron at altitudes >6 km. Dust sources in the Arctic are active during the winter and produce large amounts of particulate matter dispersed over long distances and high altitudes. HLD contributes to Arctic air pollution and has the potential to influence ice nucleation in mixed-phase clouds and Arctic amplification.

## Introduction

The Arctic surface atmosphere has undergone radical changes in past decades resulting in at least two times larger warming (~1.5 °C) than the global mean temperature change. Such Arctic warming, often referred to as Arctic amplification, is attributed to greenhouse gas feedback while short-lived aerosols act as important forcing agents as well^1–4^. The most radiation absorbing aerosols known in the Arctic atmosphere are black carbon and dark-coloured dust, but they have been also identified as strong light absorbing impurities when deposited on snow or ice^1,5–11^. Although the direct radiative forcing of aerosols in the Arctic atmosphere is estimated to be larger than indirect radiative forcing via snow feedback, early snow cover removal can result in comparatively larger climate effects^12^. The seasonality of high aerosol loadings in the Arctic is typically bimodal, with one major peak in late winter/spring and the secondary peak in autumn^3,13^.

The origin of absorbing particles is mostly attributed to long-range transport from outside of the Arctic. However, within the Arctic region there are large areas where the terrain serves as sources of dust that impact high latitudes^14,15^. We refer to these as High Latitude Dust sources (HLD). The first estimates are that all HLD sources cover >500,000 km^2^ and contribute to at least 5% of the global dust budget^14^. Iceland is the largest Arctic as well as European desert, comprised of volcanic and glacio-volcanic sediments, with high dust event frequency (>135 dust days annually) and year round occurrence^14,16–19^. Icelandic volcanic dust can be transported distances over 1000 km and it can affect large Arctic glaciated and sea areas due to its deposition on snow, ice and sea ice^9,17,20–25^. Direct aerosol measurements in Iceland have shown high Particulate Matter (PM) mass and number concentrations during dust storms *in situ*^22,26–28^ and on board of the aircraft^29^. Snow-dust storms (when dust is mixed with snow during a dust storm or deposited on snow) and some of the most extreme wind erosion events recorded on Earth have been observed and measured in Iceland, the most active HLD source in the Arctic^14,17,28,30,31^.

Measurement of the vertical distribution of aerosols is crucial for understanding the physical properties of tropospheric Arctic aerosols. However, scientific studies of airborne measurements of aerosol distribution in the Arctic are rare. Reported direct aerosol concentration measurements in vertical atmospheric profiles in the Arctic are limited to spring/summer season and low altitude of atmospheric profile (altitude ~2 km in Laakso *et al*.^32^, <3 km in Bates *et al*.^33^, <1 km in Moroni *et al*.^34^, and ~1 km in Ferrero *et al*.^35^). Winter direct measurements of Arctic aerosol profiles for the whole troposphere column in addition to the presence of the polar vortex are scarce due to operational and weather issues^36^. Vertical distribution of tropospheric aerosols during dark winter months can be retrieved, however, from satellite instruments^37,38^. Most of the direct vertical aerosol studies are focused on fine mode particles with a diameter <500 nm, but coarse particles including giant particles such as with a diameter >30 µm are capable of long range transport as well^39–43^. Aerosol studies on long-range transport of coarse and giant particles in the Arctic are generally missing.

The main objective of this study was to provide the first atmospheric vertical profiles of aerosol concentrations from balloon-borne measurements in the lower Arctic, Iceland, in winter. The purpose was to identify: (i) Icelandic dust storms and focus on understanding winter dust storms in the Arctic; (ii) number and mass concentrations with the size distributions of Icelandic dust aerosols; (iii) the nature of particles in the Icelandic lower atmosphere; and (iv) the presence of dust particles during and after snow and rain. The observations presented here provide, for the first time, a quantitative picture of dust aerosol distributions during the Arctic winter.

## Results

**Figure 1:**
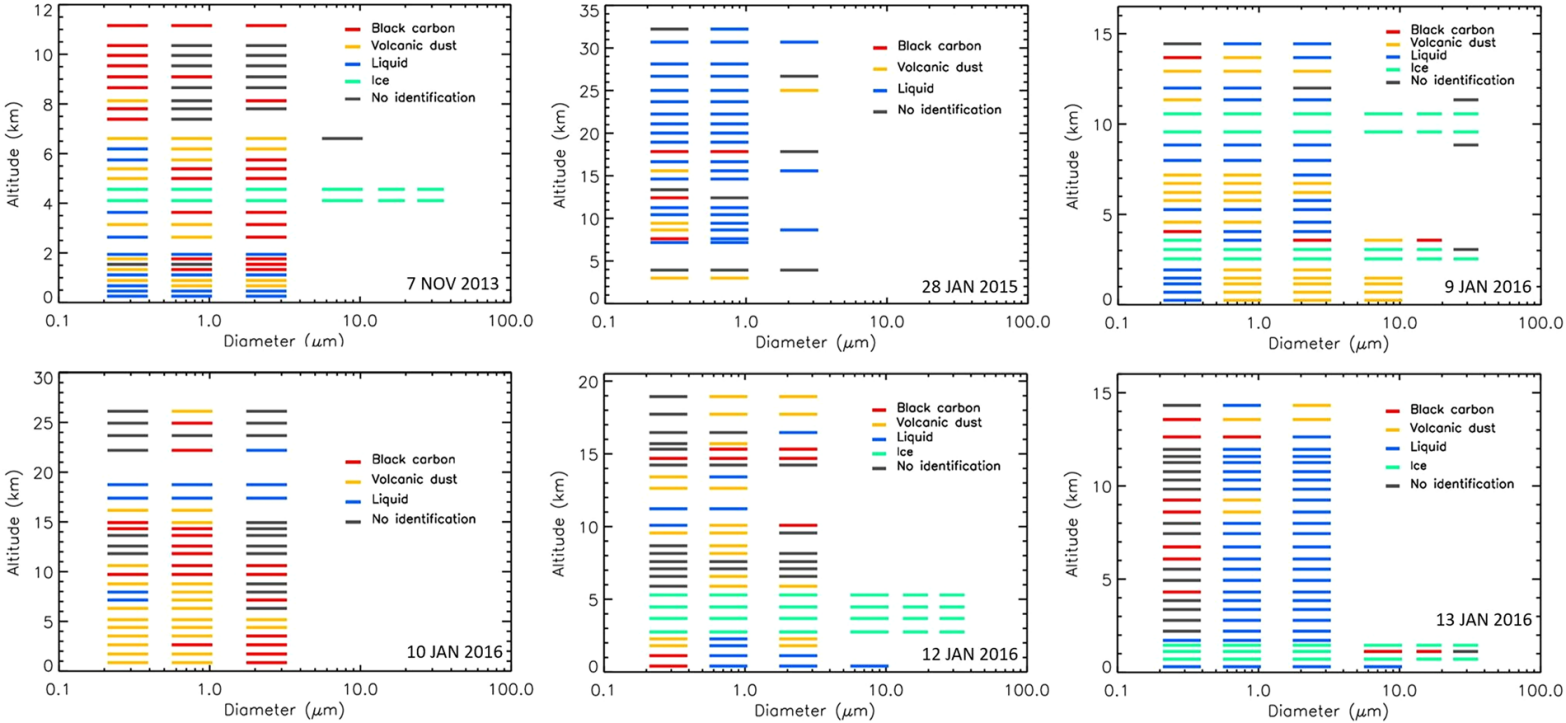
Vertical particle size distributions of the typologies for the flights on 7th November 2013, 28^th^ January 2015, 9^th^ January 2016, 10^th^ January 2016, 12^th^ January 2016, and 13^th^ January 2016. Liquid refers to transparent droplets (fog, cloud, liquid marine droplets with salt).

Vertical profile measurements to detect liquid and solid atmospheric aerosols using Light Optical Aerosol Counter (LOAC) and related meteorological parameters were conducted in south-western Iceland in 2013–2016 (for details see section Methods). Table 1 shows the description of the flights and Supplementary Fig. S1 the locations of balloon launches as well as desert and dust sources in Iceland. Different types of atmospheric aerosols were identified at several altitudes during all six flights (Fig. 1). Four out of six vertical profiles confirmed Icelandic volcanic dust, although it occurred in freezing winter or during the precipitation season: 7^th^ November 2013, 9^th^ January 2016, 10^th^ January 2016, and 12^th^ January 2016. Clean atmospheric conditions with a presence of liquid and sulphuric aerosols in the stratosphere were captured on 28^th^ January 2015 and 13^th^ January 2016^44^. In the free troposphere, mean number concentrations during the non-dust flights were <5 particles cm^−3^ for particles >0.2 µm in diameter, while mean number concentrations during the flights with dust presence increased >40 particles cm^−3^.

**Table 1:** Conditions of measurements. Date, time, location, altitude range of the flights, altitude of the tropopause, and presence of dust event of conducted flights in 2013–2016.

Date	Time of measurements (TU)	Launch location	Altitude range (km)	Tropopause (km)	Dust event
7 Nov. 2013	11:23–12:01	64.127°N, 21.911°W	0.1–11.2	8.0	Yes
28 Jan. 2015	10:43–12:48	64.346°N, 21.436°W	2.8–32.6	Not measured	No
9 Jan. 2016	15:08–16:12	63.856°N, 20.229°W	0.1–14.6	10.5	Yes
10 Jan. 2016	12:11–13:44	64.329°N, 21.652°W	0.1–26.5	11.5	Yes
12 Jan. 2016	11:33–12:47	64.337°N, 21.614°W	0.1–18.9	9.0	Yes
13 Jan. 2016	10:34–11:40	64.337°N, 21.615°W	0.1–14.6	8.0	Yes*

*Not detected by LOAC.

**Figure 2:**
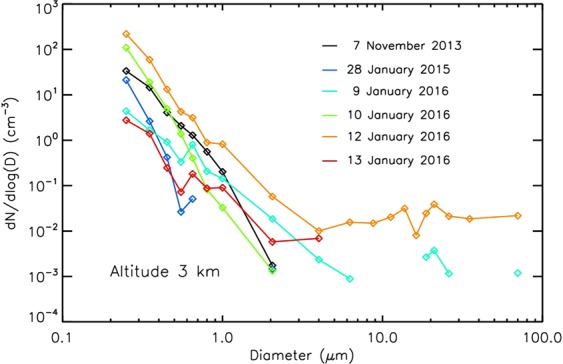
Size distribution for all the flights at an altitude of 3 km.

The concentrations of submicron particles vary between the flights, generally showing higher concentrations during dust presence than for clean condition for this Arctic region depending on the size range (Fig. 2). The shape of size distribution as shown, for example, at 3 km altitude for all the flights is not the only indicator of dust presence. The typology must be considered as in Fig. 1. Large particles >50 µm at such an altitude during the dust event on 12^th^ January 2016 refer for example to ice particles in cloud (Figs 1 and 2). The presence of dust was unambiguously detected (please see the section Methods – LOAC for detailed description) for the 9^th^, 10^th^ and 12^th^ January 2016 flights while traces of dust mixed with other aerosols were likely present during the fight on 7^th^ November 2013.

**Figure 3:**
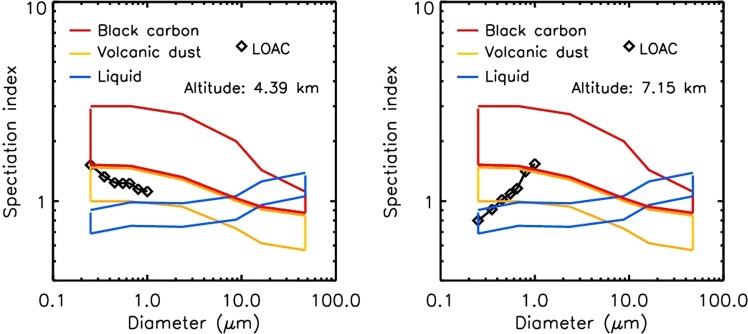
LOAC speciation index for 10^th^ January 2016 flight. Left: Inside a volcanic dust layer; right: Above the dust layer, where no identification of the particles can be done, probably because of the presence of various types of aerosols.

The best captured dust layer was recorded by Light Optical Aerosol counter (LOAC) during the flight on 10^th^ January 2016 (Fig. 1). The speciation index (described in Methods) inside a dust layer clearly determined dust typology (Fig. 3, left image) while the speciation index cannot be exactly determined for upper layer at 7 km where various types of aerosols were detected.

## Dust Events and Clean Background Conditions

### Dust Event 1–7^th^ November 2013

On 6–7^th^ November 2013, there were strong easterly winds in south-western Iceland, associated with a low-pressure system to the southwest of Iceland. Dust events occurred frequently in south-western Iceland from 1^st^ November 2013 prior to the balloon launch on 7^th^ November 2013. Surface PM_10_ concentrations in Reykjavik for the days prior to the LOAC flight were about fourfold higher than the long term average (PM_10_> 100 µgm^−3^). The main source was the Hagavatn dust source (64°27′40.0″N, 20°19′15.5″W, Supplementary Fig. S1)^17^ about 70 km from the place of the measurements and balloon launch, Reykjavik. Snow showers and rain mixed with snow occurred frequently during the day. This caused abrupt changes in the surface PM_10_ concentrations in Reykjavik^45^ with frequent peaks above 100 µgm^−3^ and drops to <10 µgm^−3^. Dust was visually observed although frequent snow-rain showers occurred.

**Figure 4:**
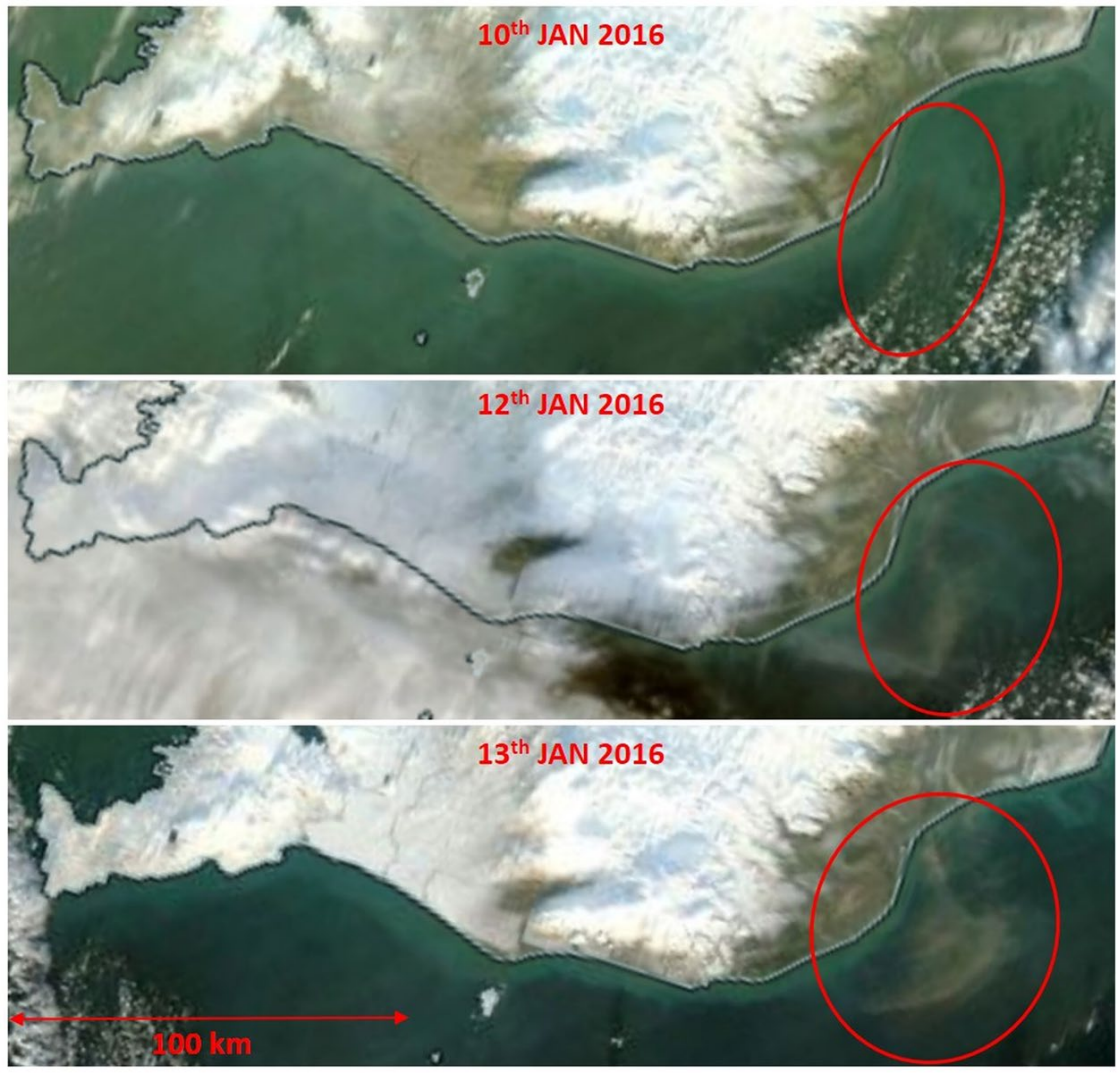
MODIS Aqua true-colour satellite images of southern Iceland from 10^th^, 12^th^ and 13^th^ January 2016 with no cloud obstruction of the dust plumes (marked red). Active dust sources are in *italic*. Image courtesy of the NASA Worldview (https://worldview.earthdata.nasa.gov/).

Figure 1 shows the vertical profile of the flight up to 11 km with the typology of captured particles. The atmosphere at an altitude of <1 km was abundant in liquid droplets, while a residual layer of suspended dust was detected at an altitude of about 900 m. The dust particles in this layer were mostly submicronic, but several particles up to 10 µm were also recorded. LOAC passed through cloud at 4 to 5 km altitude as shown by the presence of large ice particles (up to 50 µm) in typology as well as captured in the relative humidity profile. Traces of dust were also detected at an altitude of about 6.5 km. Carbonaceous particles were mainly identified during the flight at altitudes >8 km. This aerosol profile confirmed the occurrence of a small dust event with wet deposition removal of dust particles at an altitude <900 m. The total number concentration for Event 1 was 40 particles cm^−3^, while PM_10_ mass concentration was in the 5–10 µgm^−3^ range. Mean daily surface PM_10_ concentrations measured by the Environment Agency of Iceland (EAI)^45^ in Reykjavik were 24 µgm^−3^.

### Flight on 28^th^ January 2015–clean air conditions

A meso- to synoptic scale trough was over south-western Iceland, embedded in a north-easterly and larger scale low-level flow. A balloon was launched from Reykjavik in a temperature of about 0°C and winds generally from NW to N, but only 5–10 ms^−1^ throughout the troposphere. The total number concentration for non-polluted air was of about 1–5 particles cm^−3^ and the PM_10_ mass concentration was of about 2–5 µgm^−3^. Low surface PM_10_ concentrations (daily mean of 14 µgm^−3^) did not exceed 25 µgm^−3^ at the aerosol monitoring station in Reykjavik.

### Dust Event 2–9^th^ January 2016

The beginning of January 2016 can be described as a period of unusually cold conditions when snow cover exceeded 90% of the country and a strong polar vortex developed above Iceland^46^. Surface temperatures were below 0°C and dropped to −70 °C at altitudes above 10 km.

On 9^th^ January 2016, there was a deep low moving to the east and south of Iceland and there were strong low-level winds from the east, northeast over Iceland. Most of the dust sources were covered with snow, but recently exposed sediments after subglacial eruption ‘Skaftárhlaup’ in Southern Iceland were frequently suspended as captured by MODIS satellite (Fig. 4). Visible dust plumes over the ocean were mostly transported south- and southwest-wards of Iceland during strong north-easterly winds. The balloon was launched from the area close to the village of Hella in southern Iceland (Supplementary Fig. S1). The only operating instrument in Reykjavik^45^ (about 250 km northwest from the source area) captured increased PM concentrations (PM_10_ up to 150 µgm^−3^ and PM_2.5_ up to 110 µgm^−3^) on 9^th^ (mean PM_10_ = 19 µgm^−3^ with max >40 µgm^−3^) and 10^th^ January 2016. Figure 1 show the vertical profile on 9th January 2016 up to 14 km and typology of captured particles. Two dust layers were detected by LOAC, one from the ground to an altitude of 2 km, and another between 6 and 7 km. Clouds were crossed at altitudes of 3–4 km, and cirrus at altitudes of 9–11 km. The highest concentrations of 70 cm^−3^ were detected at an altitude of 6.5 km, giving a relatively low PM_10_ mass concentration of about 5 µgm^−3^, mainly showing the presence of submicron particles. An additional thin aerosol layer can be seen in the lower stratosphere at about 13km. This could be related to the descent of solid particles from the polar vortex.

### Dust Event 3–10^th^ January 2016

Dust event 3, on 10^th^ January 2016, was captured well during the flight, showing the highest dust rates in the troposphere from all the flights (Fig. 1). The balloon was launched from Hvalfjördur Bay in south-western Iceland (Supplementary Fig. S1). A trough formed over western Iceland and the wind profile (Supplementary Fig. S2) is on the eastern edge of the trough, featuring southerly and south-easterly winds of 5 ms^−1^ in the lower and middle troposphere. These winds transported dust particles westwards from the source at Skaftarhlaup, as shown in Fig. 4. Wind profiles obtained from the LOAC flight show surface winds arriving from the directions where increased PM_10_ concentrations were measured (Reykjavik, mean surface PM_10_ = 67 µgm^−3^ with 30-min max >150 µgm^−3^). There was a wind direction change (linked with wind velocity increase) at altitudes between 1–2 km showing winds arriving from the area of suspended jökulhlaup sediments.

The speciation index mainly indicated dust particles in the whole troposphere for particles <5 µm (Figs 1, 3). The larger particles were attributed to “black carbon” typology at altitudes <5 km. These aerosols were either carbonaceous particles or (aggregates of) volcanic dust particles that are highly porous and strongly optically absorbing. For altitudes above 5 km in the troposphere, the typology results are more scattered although optically absorbing particles seem to dominate. The highest concentrations were at an altitude of 2 km with about 250 particles cm^−3^ while the mass concentration was in the 10–15 µgm^−3^ range, showing a lack of large particles.

### Flights during the Dust Event 4-12-13^th^ January 2016

On 12 January 2016, there were lows traveling to the south and southeast of Iceland giving strong north-easterly low-level winds over Iceland itself. Satellite images in Fig. 4 show dust events in southern Iceland on 12^th^ and 13^th^ January 2016. Surface concentrations in Reykjavik were, however, low (mean daily PM_10_ <13 µgm^−3^) due to the winds arriving from different directions than the active dust sources (Supplementary Figs S1, S2). LOAC was launched in Hvalfjordur, about 200 km northwest from the dust source, on 12^th^ January 2016 and it did capture a small amount of dust in the troposphere. The mean number concentration detected by LOAC in the free troposphere was about 20 particles cm^−3^, while total mass concentration was 2–5 µgm^−3^. Wind profiles on 12^th^ January in Supplementary Fig. S2 show a low-level north-easterly jet of about 15 ms^−1^, turning to weaker north-westerly winds at middle tropospheric levels. On 13^th^ January 2016, the low-level NE winds were down to 8–10 ms^−1^, with westerly 5–10 ms^−1^ at middle-tropospheric levels. Surface wind speeds were considerable higher on 12^th^ January than 13^th^ January 2016. No dust was detected on the 13^th^ January flight, with the presence of only about 5 particles cm^−3^. Daily mean PM_10_ concentrations from the station in Reykjavik were 10–13 µgm^−3^.

## Discussion

Six successful winter balloon launches were conducted in Iceland in 2013–2016, despite the harsh outside conditions with winds at the highest level possible for such a launch. Atmospheric profiles (up to 32 km) of aerosol distributions were measured for both clean Arctic air and air heavily polluted by natural volcanic dust from the local Arctic (Icelandic) deserts and fresh sediments from Skaftárhlaup frequently suspended after a subglacial eruption in September 2015. The vertical profiles with dust concentrations at different altitudes do not show evident dust layers as detected previously with LOAC in Saharan dust plumes^42^. Saharan dust layers are unambiguously characterized by the presence of a coarse mode of particles of at least several micrometres^39–43^. Measured dust layers of local, glacially reworked, volcanic materials in Iceland are often comprised of submicron sized particles, resulting in low mass concentrations^26,28^. Such volcanic dust particles can be precisely identified by combining particle counting and optical typology measurement methods, as obtained with LOAC. High proportions of submicron particles have been observed during previous *in situ* measurements of Icelandic dust storms^26,28^. The highest number concentrations of dust particles (0.3–10 μm) were in the size range 0.3–0.337 μm in a dust storm in 2013. High PM_1_ concentrations were also measured during two dust storms in 2015, where the PM_1_/PM_10_ mass ratios ranged from 0.34–0.63. Moreover, submicron/supermicron ratios of 0.5 were calculated from particle number concentrations measured during the flight of aircraft inside Icelandic dust storm in 2017^47^. Such high ratios are usually reported in polluted urban areas in Europe or Asia, rather than during natural dust air pollution.

All winter dust events presented here were either captured by the satellite or reported from the surface PM_10_ measurements at the Reykjavik EAI station (Fig. 4). The long-term frequency of dust events in the southern part of Iceland showed that about half of reported dust events in 1949–2011 occurred during winter or sub-zero temperatures^16^. Vignelles *et al*.^48^ observed dust plumes from desert surfaces in northeast Iceland in January 2015. Winter dust storm occurrence in Arctic areas such as Iceland is important because of the interaction of dust and cryosphere. Snow Dust Storm phenomena occur in Iceland while albedo reduction and melting of the glaciers are caused due to light absorbing impurity, dark volcanic dust, in Iceland^10,28,49^ and in the Arctic^9,24^.

Quantifying the aerosol distributions in high atmospheric profiles gives an overview of air quality for clean background Arctic conditions as well as polluted air conditions. The number concentrations during the cleanest conditions were <5 particles cm^−3^ in the 0.2–100 µm size range. It is difficult to provide comparisons with our measurements in clean background conditions due to the diversity of instruments and particle size ranges for atmospheric profile measurements reported in the literature. Kupiszewski *et al*.^50^ reported median concentrations of particles with diameter D >0.3 µm of about 1 particle cm^−3^ within 1 km a.g.l. in the high Arctic. Concentrations of D_0.5–1_ particles ranged from 1–12 cm^−3^ in coastal Norway^51^. The Arctic aerosol concentration of submicron particles (D_0.3–0.8_) is generally very low, about 1 cm^−3^ within 1 km a.g.l., but it can increase by several factors, sometimes by as much as an order of magnitude, at certain heights within the free troposphere^52^. Median monthly D_0.2–0.5_ concentrations range between about 1–50 cm^−3^ in the Canadian Arctic and about 5–70 cm^−3^ in Svalbard, Norway (Mt. Zeppelin)^13^. Background surface concentrations of D_0.26–1.2_ was 15–38 cm^−3^ and D_1.2–32_ was about 0.01–0.3 cm^−3^ in Svalbard^35^. Concentrations of D_0.25–32_ were 0.03–0.2 cm^−3^ during two flights in Svalbard^34^. Increased ground concentrations of D_0.5–20_ were up to 10 particles cm^−3^ in Svalbard in April 2011 due to long range transport of Icelandic dust^24,34^. This shows that there is a large diversity in aerosol number concentrations in the Arctic region and our measurements are in good agreement with most of them.

Number concentrations during dusty conditions ranged from 40–250 particles cm^−3^ in the 0.2–100 µm size range. Concentrations >200 particles cm^−3^ are typical for Saharan dust outbreaks^42,53^. A winter dust storm was measured in Iceland on 25^th^ February 2007, when a Passive Cavity Aerosol Spectrometer Probe (optical counter for particles of 0.1–3 μm diameter) was on board the aircraft^29^. About 100–300 particles cm^−3^ were measured at 400 and 700 m altitudes inside the dust plume while 10–50 particles cm^−3^ were measured at an altitude of 1900 m. Large particles ~20 µm were collected inside Icelandic dust storm at 320 m altitude in 2017^47^. This shows that dust events cause considerable air pollution as well as influence ice nucleation in mixed-phase clouds in the Arctic^47^.

Surface PM_10_ concentrations during dust events in Reykjavik were about six times higher than non-dusty conditions with 30-min PM_10_ peaks >100 µgm^−3^ on 7^th^ November 2013 and 10^th^ January 2016. On 9^th^ January 2016, twice as high PM_10_ concentrations than non-dusty conditions were reported. Clean air with daily PM_10_ mean concentrations of about 10–13 µgm^−3^ were measured in Reykjavík on 12–13^th^ January 2016, but satellite images captured well-developed dust storms in southern Iceland (Fig. 4). The reason for such low concentrations was the wind direction at the measurement site, which changed to northerly and north-westerly winds instead of easterly and south-easterly winds from the active dust sources.

We examined aerosol profiles obtained by the Cloud-Aerosol Lidar and Infrared Pathfinder Satellite Observations (CALIPSO) for the dust events. It was, however, only the launch on 13^th^ January 2016 from all the LOAC flights when the satellite was passing over the exact location of the measurements. The aerosol profiles from the other days are related to the ocean areas outside Iceland. On 7^th^ November 2013, CALIPSO detected marine and dusty marine aerosols at <1.5 km altitude, dust in three layers (at about 3 km, 5–6 km and 8–9 km) and aerosols attributed to ‘elevated smoke’ at about 8 km above the sea about 200 km south of Iceland. This is in agreement with measurements provided by LOAC which identified: (i) marine aerosols and hydrated dust particles at <1 km altitude; (ii) a thin dust layer at about 6 km; and (iii) carbonaceous particles >8 km. On 28^th^ January 2015, CALIPSO captured marine aerosols at <500 m and a clean profile up to 22 km altitude over the sea south of Iceland (distance about 150 km). Many aerosol types at several altitudes were detected by CALIPSO west of Iceland on 9^th^ January 2016. Aerosols attributed to “polluted dust” and”elevated smoke” were detected at altitudes of 3–4 km and 5–7 km, while dust was at 4–5 km, around 7 km, and 10–11 km (250 km west of Iceland). LOAC measured dust aerosols <2 km, 6–7 km, and at about 13 km. The aerosol signatures in the higher altitudes were comparable to what LOAC captured.

On 10^th^ January 2016, the early morning CALIPSO profile above Iceland showed the presence of marine and marine dusty aerosols at <1.5 km, “elevated smoke” at 2–3 km, and dust layer at 9–11 km south-easterly of Iceland. Polar Stratospheric Clouds (PSC) were shown at 17–18 km. The afternoon passage was over NE Iceland and related the aerosols to “polluted continental or smoke” origin at altitude <2 km. A mixture of dust and polluted dust particles was shown at 6–8 km. PSC were at 20–23 km. The speciation index of aerosols detected by LOAC also classified the particles as black carbon at altitudes <5 km for particles >5 µm. The fine particles were, however, dust. LOAC was flying downwind through the dust plume spotted on the satellite indicating why both datasets are in good agreement. Both instruments have shown the presence of dust particles in higher altitudes up to 10 km.

**Figure 5:**
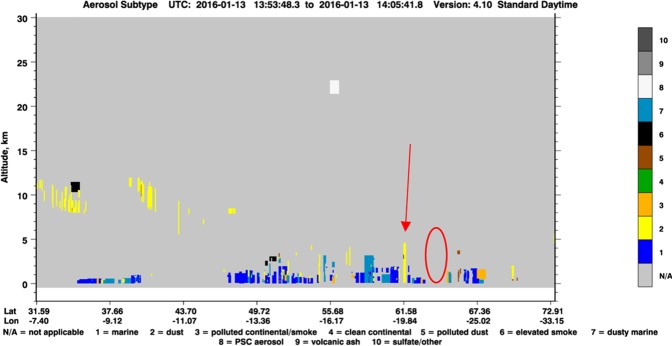
CALIPSO cross sections of 532 nm total attenuated backscatter and the vertical feature mask for the overpass of Iceland on 13^th^ January 2016. LOAC flight marked with red circle. The relationship between the dust column marked with red arrow and the dust plume captured in Fig. 4 is questionable (image from courtesy of the NASA/CALIPSO system, https://www-calipso.larc.nasa.gov/).

The CALIPSO passage was too far from Iceland on 12^th^ January 2016, while it passed over near the location of the LOAC flight on 13^th^ January 2016 at about the same time of the launch. CALIPSO observations showed a clean profile with no aerosol detected when passing over the location of the LOAC flight and further south and southeast (Fig. 5). It suddenly detected an aerosol signature attributed to dust particle from the ground up to 5 km south of Iceland at about 62N 20W (100 km of the coast). A profile of about 4 km from the ground with a dusty marine signature was detected further south and east. The relationship between the CALIPSO observation and the dust plume captured by satellite (Fig. 4) is questionable. It provides a possible explanation of why LOAC did not measure many particles during this particular flight, despite the fact that a dust storm was occurring about 200 km from the balloon launch. Although CALIPSO may be underestimating optical depths for Saharan dust^54^, it seems to detect well dark volcanic Icelandic dust over sea areas as captured by LOAC. The vertical aerosol measurements in the Arctic from LOAC as well as from CALIPSO have shown that dust can be found at high altitudes, such as up to several km. Dust from the sources located above 60°N was found to reach altitudes up to 8 km in winter emphasizing that Icelandic dust is able to reach the upper Greenland ice sheet^15^.

It is known from Saharan vertical dust measurements that dust events aged over 12 h since uplift contain giant particles up to 5 km altitudes^55^. Vertical velocities were over ±30 cms^−1^ in all cases, and up to ±80 cms^−1^ within the Saharan air layer (SAL), >200 cms^−1^ within the convective boundary layer, and frequently over 50 cms^−1^ up to 5 km altitude. High levels of atmospheric turbulence could, therefore, sustain transport of dust particles for longer than expected by gravitational sedimentation. Another mechanism could be solar absorption by the dust particles, possibly generating convection and daytime vertical mixing within the SAL^56^. Darker particles in the solar spectrum could increase convection and mixing. Optical properties of Icelandic volcanic dust are similar to black carbon with a spectral reflectance of a nearly black body (0.03)^7,8^.

The dust source of the storm on 7^th^ November 2013 was Hagavatn dust hot spot, about 70 km NE of Reykjavik on the southern edge of the glacier Langjokull^17^. HYSPLIT back trajectory at 900 m calculated moving air parcels from the dust source^57^. The trajectory at 6000 m could explain the small layer of dust detected by LOAC at this altitude. Air parcels travelled around Iceland for about 144 hours, originating from an altitude of about 1000 m at the time when dust plumes were visible on satellite images (2^nd^ and 1^st^ November 2013). This could indicate that the origin of the thin layer at 6 km could also have been Icelandic dust. HYSPLIT forward trajectory analysis from 10^th^ January 2016 shows uplift of air parcels from the area of suspended sediments visible on the satellite. The 72 h-trajectories pass the area of Hvalfjordur on 12^th^ January at altitudes above 5 km and marine areas south of Iceland at altitudes 4–5 km on 13^th^ January 2016. This could indicate that LOAC captured traces of the jokulhlaup sediments on 12^th^ January 2016, but they were already suspended on 10^th^ January 2016. It is, however, not clear if this was the material captured by LOAC and CALIPSO at such altitudes. We have not found any evidence that other dust sources were activated during the experiments.

Important sources of additional aeolian material are frequent volcanic eruptions (one eruption each 3–4 years on average) and sediments from glacial outburst floods, so called jokulhlaups, after subglacial eruptions. Jokulhlaups occur in Iceland more frequently than explosive volcanic eruptions^58^. Large jokulhlaups can bring >5 million tonnes of very fine deposits (grain size <0.05 mm) available for suspension every year. An example of such large event was Skafta jokulhlaup from September 2015. The sediments were frequently suspended in November and December 2015. Figure 4 reveals that the main dust hot spots were covered with snow while the jokulhlaup sediments were lifted by winds on the south coast of Iceland.

## Conclusion

The Arctic includes important sources of natural dust, which is directly affecting the Arctic atmosphere and cryosphere. Iceland is the largest desert in the Arctic (and Europe), covering 44,000 km^2^ including the most active dust areas, so called dust hot spots, located in the vicinity of glaciers. This study provides evidence that HLD sources, such as those in Iceland, are actively producing dust aerosols, causing winter Arctic dust storms and impairing air quality, including at high altitudes. Icelandic dust storms can increase particle number concentrations at an altitude of 2 km to the same values as measured during Saharan dust plumes. Detected volcanic dust particles reworked by glacial processes are, however, smaller in size than crustal dust from the Sahara. Contrarily, atmospheric profiles during clean conditions showed <5 particles cm^−3^ for the particles >0.2 µm in diameter. This is similar to what has been reported from other Arctic locations. More vertical aerosol measurements of HLD storms are needed, although it is a difficult task due to the harsh conditions. Having monitoring and better understanding of dust storms from Iceland (mainly in terms of particle size distributions, concentrations with altitude, transport and deposition) is important for better assessment of the role of volcanic dust in the Arctic climate and cryosphere. HLD does not only cause considerable air pollution close to the surface, it can also significantly influence low-level clouds in the Arctic serving as ice nucleating particles. Dust has been recognized as important climate driver causing snow darkening and melting in Polar Regions in the IPCC report in 2019^59^. More research is urgently needed on the HLD sources and impacts on cryosphere and atmosphere.

## Methods

Vertical profile measurements were conducted in south-western Iceland in 2013–2016. Table 1 shows the date, location (Supplementary Fig. S1), altitude range of the flights, altitude of the tropopause, and presence of dust event. It should be noted that the polar vortex was present over Iceland during the January 2016 flights and that the tropopause was not well marked. The number of winter profiles was limited due to loss of most of the instruments during the descending phase in harsh weather. As a result, six flights in winter using weather balloons were successful.

**Figure 6:**
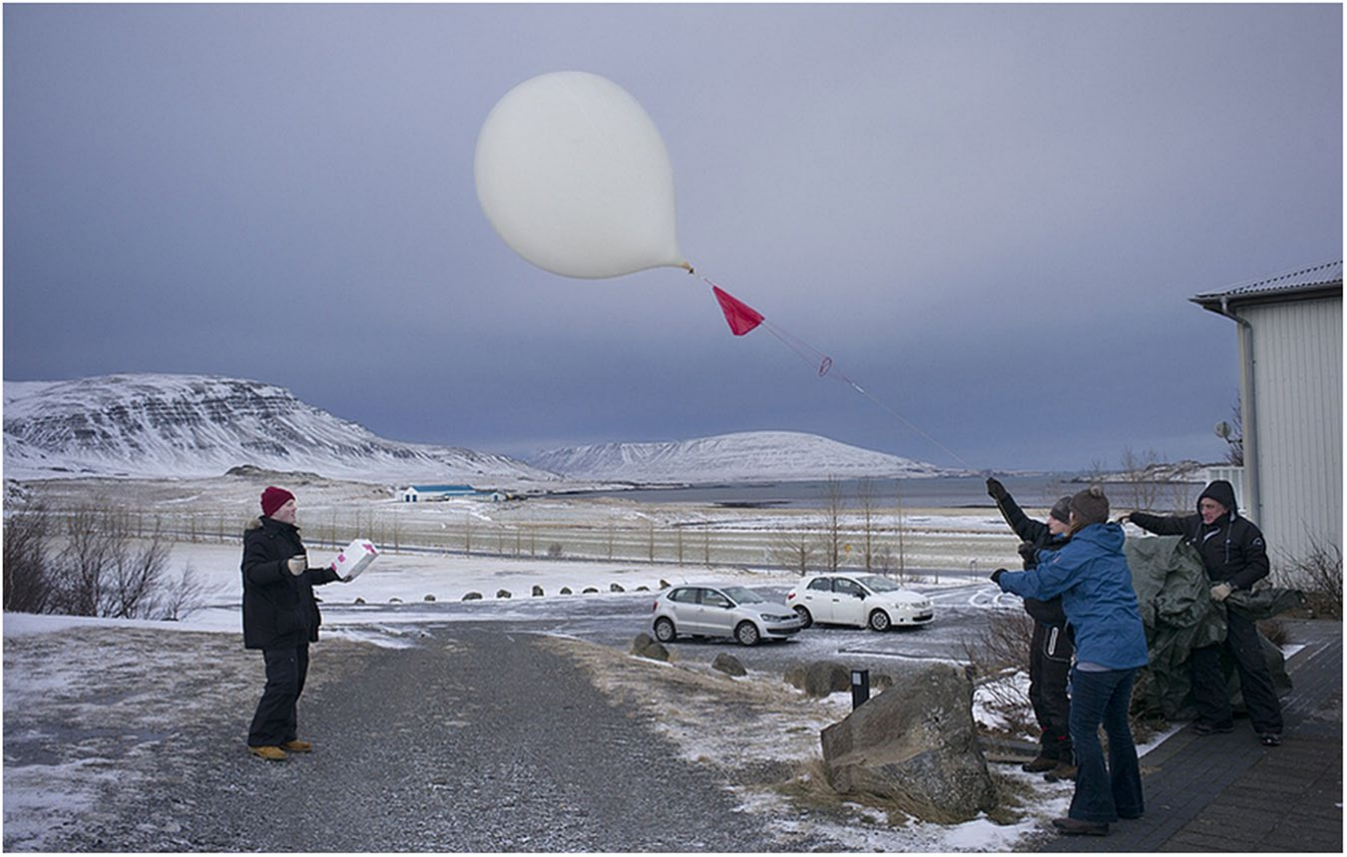
Launch of LOAC during strong winds in Hvalfjordur bay, West Iceland, on 12^th^ January 2016 (Photo by RAX, Ragnar Axelsson).

A novel instrument, Light Optical Aerosol Counter (LOAC), was used to measure the aerosols size number distributions and concentrations, to estimate their typology, and to provide the basic meteorological elements up to an altitude of 11.2–32.6 km, depending on the burst altitude of the balloon or telemetry loss (the data are sent in real time). LOAC is an easy-to-launch instrument with the possibility to conduct the flight with ground wind speeds up to 15 ms^−1^, which is suitable for harsh Icelandic winter conditions. Figure 6 shows an example of LOAC launch from Hvalfjörður on 12^th^ January 2016. This paper focuses on the measurements in the troposphere, although some profiles include stratospheric measurements, and on the different types of aerosols detected with LOAC.

### LOAC instrument

Light Optical Aerosol Counter (LOAC) is an aerosol counter/sizer that provides concentration measurements for 19 size classes in the 0.2–100 µm size range and an estimate of the typology of the aerosols^60^. LOAC has been carried on several types of balloons, mainly weather balloons^53^. The weight of the gondola, including the instrument, the alkaline batteries and the telemetry system is about 1 kg. The aerosols are injected into a laser beam using a small pump. The measurements are conducted at two scattering angles: the first angle is at around 12°, where the light scattered by non-spherical particles is dependant strictly on the size of the particles^61^, while the second angle is around 60°, where the scattered light is strongly dependant on the refractive index of the particles^61,62^. The 12° angle measurements are used to retrieve the size distribution of the particles, and the combined measured signals at both angles are used to calculate the LOAC “speciation index” for the typology of aerosols. Obtained values of specification index are compared with reference “speciation zones” determined by LOAC in the laboratory for typical samples such as black carbon, liquid droplets, and ice particles. In case of Icelandic volcanic dust, a specific “speciation zone” has been established in the laboratory using different samples of volcanic dust and ash collected in Iceland. The speciation index is calculated for each size class. The typology is determined for six size “super-classes” centred on 0.3 µm, 0.8 µm, 2.5 µm, 8.0 µm, 16.5 µm, and 30 µm that combine the speciation index of three consecutive size classes. The typology indicates if a specific nature of particles dominates in the sampled air mass; no indication can be retrieved in the case of a heterogeneous medium. The unambiguous presence of dust/ash is stated if “dust/ash” typologies are retrieved for at least three consecutive super-classes.

Concentration measurement uncertainty is of ±20% for the concentrations higher than 10 particles cm^−3^. The uncertainty increases to about ±30% for submicronic particle concentrations higher than 1 particle cm^−3^, and to about ±60% for concentrations smaller than 10^−2^ particle cm^−3^. Additional uncertainties come from the size calibration, which are of ±0.025 µm for particles smaller than 0.6 µm, 5% for particles in the 0.7–2 µm range, and of 10% for particles greater than 2 µm. Accurate detection of submicronic particles and larger particles by LOAC was validated during numerous inter-comparison sessions with different instruments^42,59,60^. LOAC performs measurements every 10 seconds, typically over a session of 10 or 15 minute, while a one-minute automatic self-calibration is performed to control electronic noise and to correct the effect of temperature variation on the electronics in-between the periods. Detailed information on LOAC can be found in Renard *et al*.^60^.

### Balloon-borne dust measurements

**Figure 7:**
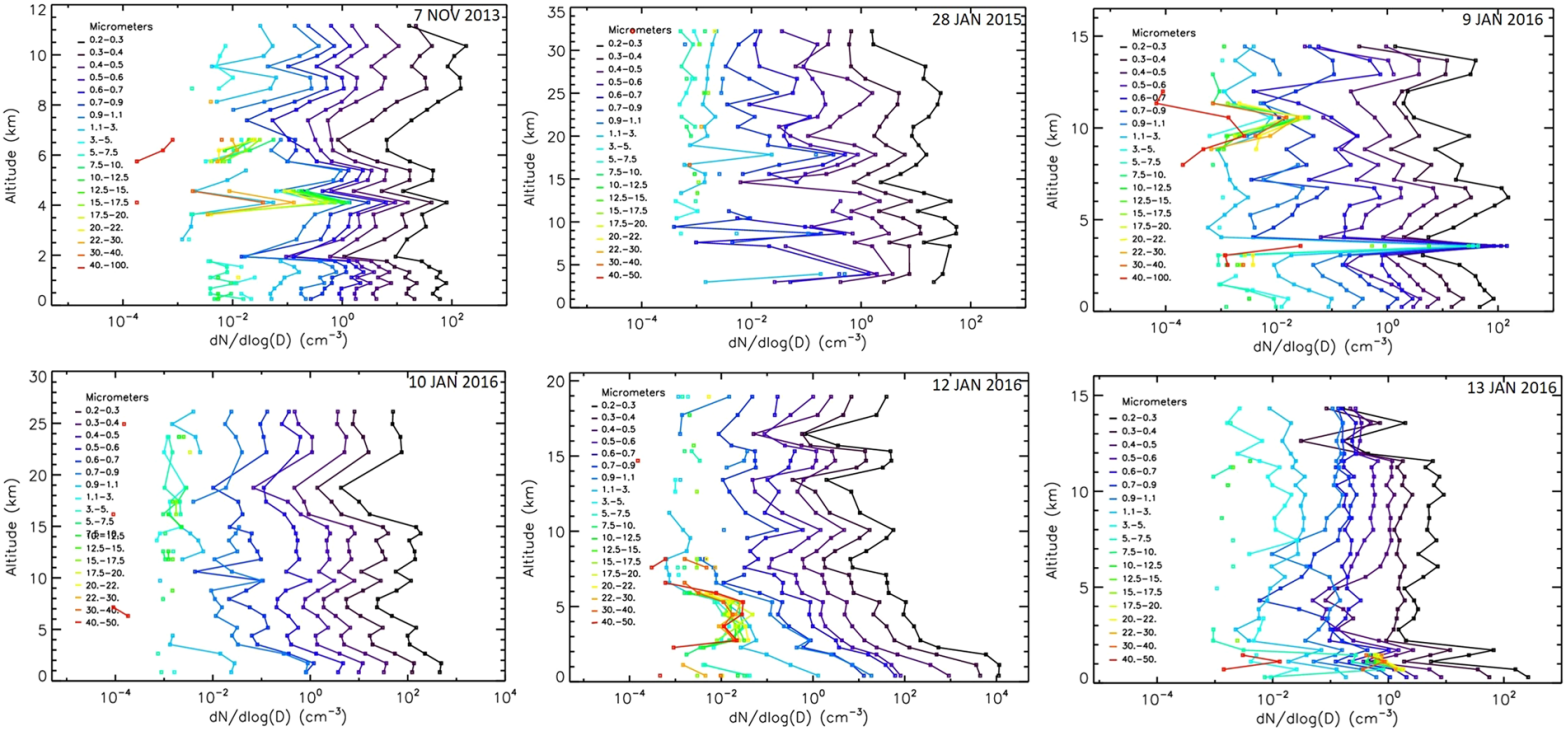
Particle size distributions at different altitudes for the flights on 7th November 2013, 28^th^ January 2015, 9^th^ January 2016, 10^th^ January 2016, 12^th^ January 2016, and 13^th^ January 2016.

Six vertical high-altitude profiles obtained with LOAC at 19 particle size classes are presented in Fig. 7. The peaks in the aerosol concentrations with the altitude can be related to different types of aerosol such as marine aerosols, clouds, pollution particles, or ice (Fig. 1). Clouds of liquid droplets and/or ice particles are characterized by the presence of larges particles (>10 µm), as shown in green, yellow, and red in Fig. 7, for example, for the flight on 12^th^ January 2016. Micron and submicron liquid aerosols are present from the ground to the stratosphere (Figs 1, 7).

### Meteorological conditions during the flights

Five of six conducted flights (Table 1) occurred during dust events from different dust sources in Iceland as described in Arnalds *et al*.^17^ (Supplementary Fig. S1). Meteorological conditions controlled the main characteristics of single dust events. Event 1 (7^th^ November 2013) was a small dust storm with surface winds of about 8 ms^−1^, precipitation of 1.5 mm, and a surface temperature of about 3 °C. Events 2, 3 and 4 (on 9^th^, 10^th^, and 12^th^ January 2016) were moderate-severe dust storms with surface winds of about 15 ms^−1^ (Supplementary Fig. S2) and surface temperatures from 0 °C to −5 °C. The dust event on 13^th^ January 2016 was not captured by LOAC. There was no dust present in the atmosphere in Iceland on 28^th^ January 2015.

### Cloud-Aerosol lidar and infrared pathfinder satellite observations (CALIPSO)

Detection of aerosols in high latitude regions such as Iceland is complicated for most of the satellite-based passive remote sensing instruments due to frequent cloud cover, darkness in winter, and bright surfaces. To be able to compare results from LOAC we used data from Cloud-Aerosol Lidar and Infrared Pathfinder Satellite Observations (CALIPSO) around Iceland. CALIPSO can detect aerosols in clear sky conditions, beneath thin cloud layers, as well as at night, and it provides high altitude vertical profiles of atmospheric aerosol distributions^63^. The CALIPSO satellite carries the Cloud-Aerosol Lidar with Orthogonal Polarization (CALIOP) instrument that operates at two wavelengths (532 nm and 1064 nm) and it provides continuous observation with attenuated backscatter covering the entire globe. The vertical distribution of different types of aerosols from LOAC flights is compared to CALIPSO cross sections of 532 nm total attenuated backscatter and the vertical feature mask for the overpass around Iceland. Data were obtained from the NASA Langley Research Center Atmospheric Science Data Center (http://wwwcalipso.larc.nasa.gov/products/lidar/browse_images/show_calendar.php). Comparisons of CALIPSO products with different aerosol observation networks have shown good agreement, but the CALIPSO retrievals may lead to an underestimation of dust optical depths^54,64^.

## Supplementary information

Supplementary Information

## Data Availability

The LOAC data that support the findings of this study are available at the ESPRI Data Centre of the French Atmosphere Infrastructure (AERIS, http://cds-espri.ipsl.fr/etherTypo/index.php?id=1699&L=1). National Aeronautics and Space Administration (NASA) supports an open data policy and encourage publication of imagery from Worldview as used in Fig. 4 (https://worldview.earthdata.nasa.gov) as well as from CALIPSO as used in Fig. 5 (https://www-calipso.larc.nasa.gov).
