# Supplementary material for: Vertical distribution of aerosols in dust storms during the Arctic winter
Source: Sci Rep. 2019 Nov 6;9:16122. doi: 10.1038/s41598-019-51764-y (PMC6834589; doi:10.1038/s41598-019-51764-y)
Supplement: Supplementary file 1 — Supplementary Information [file 41598_2019_51764_MOESM1_ESM.pdf]

Vertical distribution of aerosols in dust storms during the Arctic winter

Pavla Dagsson-Waldhauserova[1,2,*], Jean-Baptiste Renard[3], Haraldur Olafsson[4,5], Damien Vignelles[3], Gwenaël Berthet[3], Nicolas Verdier[6], Vincent Duverger[3]

[1]Agricultural University of Iceland; Faculty of Environmental Sciences, Hvanneyri; Borgarnes; IS 311; Iceland; *pavla@lbhi.is
[2]Czech University of Life Sciences Prague; Faculty of Environmental Sciences; Department of Ecology; Prague; 160 00; Czech Republic
[3] LPC2E-CNRS, 3A avenue de la recherche scientifique, 45071 Orléans cedex 2, France
[4]University of Iceland; Department of Physical Sciences; Reykjavík; Iceland; IS 101
[5] Icelandic Meteorological Office, Reykjavik, Iceland
[6] Centre National d 'Etudes Spatiales, 18 avenue Edouard Belin, 31055 Toulouse cedex, France

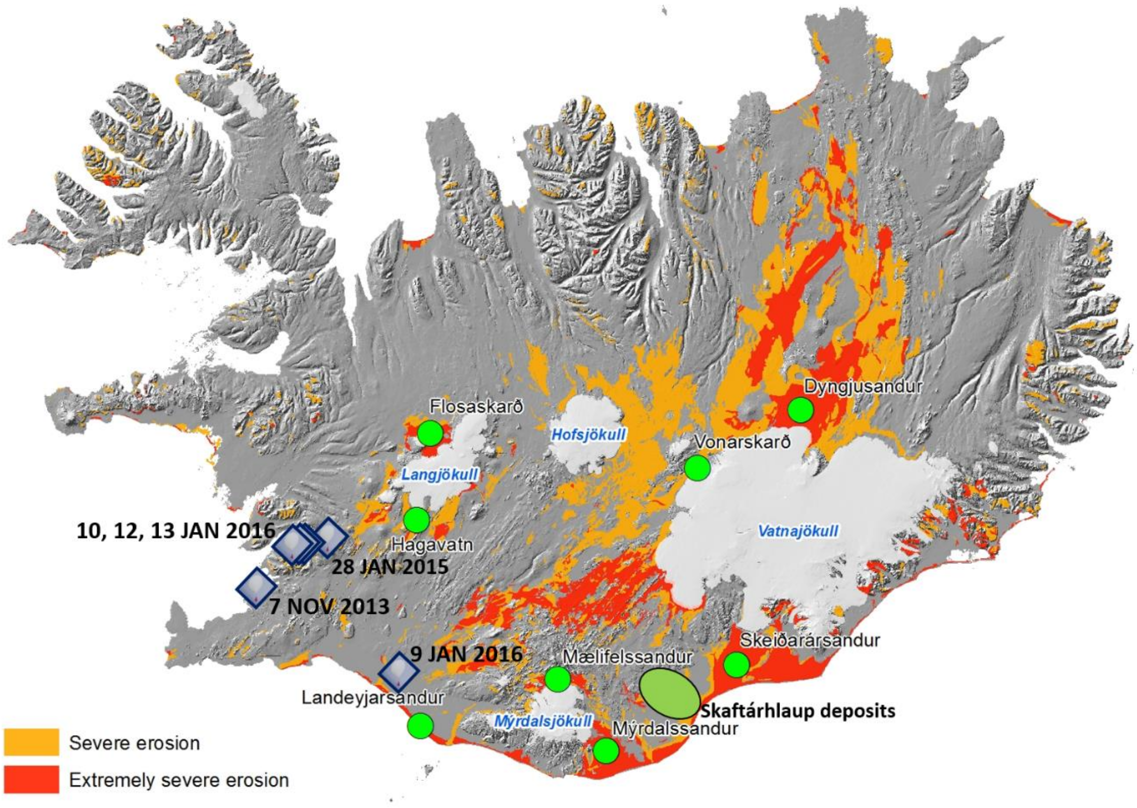

Figure S1| Locations of LOAC balloon launches (squares), dust hot spots (circles) and Skafta jokulhlaup deposits (ellipse). Sandy deserts in Iceland are marked according to their erosion severity (yellow: severe erosion; red: extremely severe erosion). Map: AUI Soil Erosion Database, prepared by Sigmundur Helgi Brink and Olafur Arnalds, Agricultural University of Iceland.

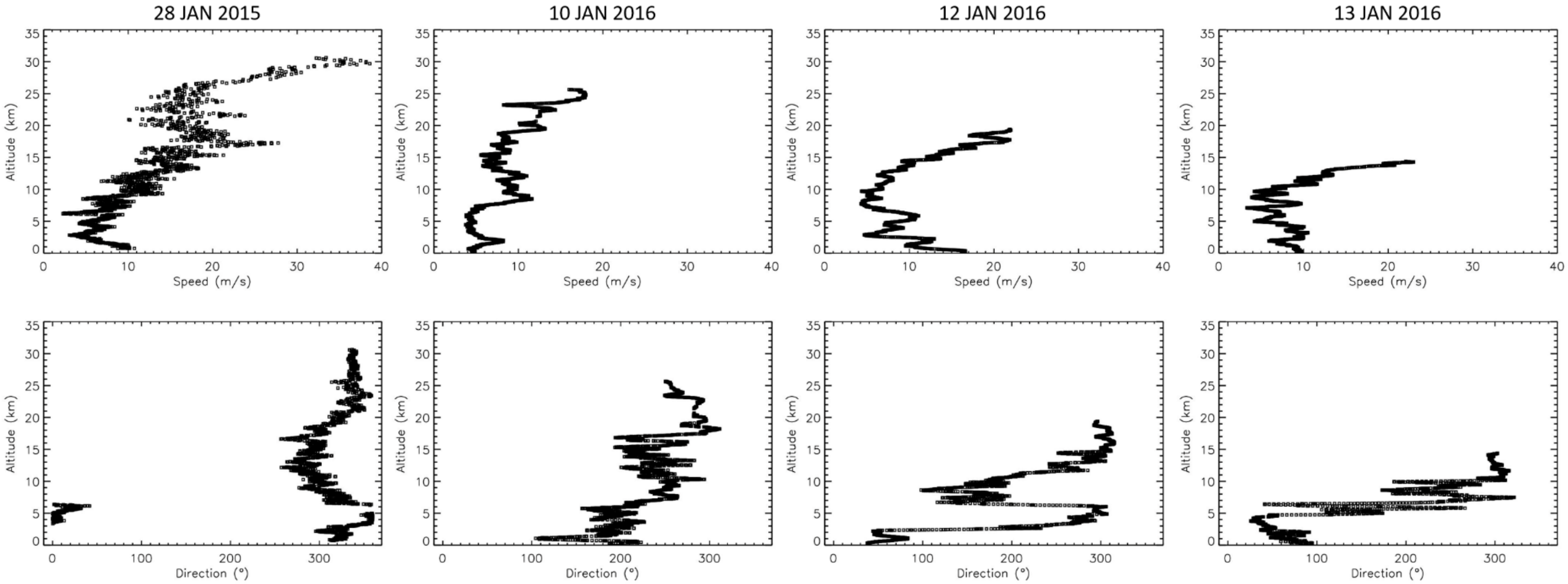

Figure S2| Vertical profiles of wind speed (upper row) and wind direction (lower row) obtained during LOAC launches on 28th January 2015 and 10th, 12th and 13th January 2016.
